# Aggregation Pattern Transitions by Slightly Varying the Attractive/Repulsive Function

**DOI:** 10.1371/journal.pone.0022123

**Published:** 2011-07-20

**Authors:** Zhao Cheng, Hai-Tao Zhang, Michael Z. Q. Chen, Tao Zhou, Najl V. Valeyev

**Affiliations:** 1 State Key Laboratory of Digital Manufacturing Equipments and Technology, Huazhong University of Science and Technology, Wuhan, People's Republic of China; 2 The Key Laboratory of Image Processing and Intelligent Control, Department of Control Science and Engineering, Huazhong University of Science and Technology, Wuhan, People's Republic of China; 3 Department of Electrical and Computer Engineering, Temple University, Philadelphia, Pennsylvania, United States of America; 4 Department of Mechanical Engineering, The University of Hong Kong, Pokfulam, Hong Kong SAR, People's Republic of China; 5 School of Automation, Nanjing University of Science and Technology , Nanjing, People's Republic of China; 6 School of Computer Science and Engineering, University of Electronic Science and Technology of China, Chengdu, People's Republic of China; 7 Centre for Molecular Processing, School of Engineering and Digital Arts, University of Kent, Kent, United Kingdom; University of Maribor, Slovenia

## Abstract

Among collective behaviors of biological swarms and flocks, the attractive/repulsive (A/R) functional links between particles play an important role. By slightly changing the cutoff distance of the A/R function, a drastic transition between two distinct aggregation patterns is observed. More precisely, a large cutoff distance yields a liquid-like aggregation pattern where the particle density decreases monotonously from the inside to the outwards within each aggregated cluster. Conversely, a small cutoff distance produces a crystal-like aggregation pattern where the distance between each pair of neighboring particles remains constant. Significantly, there is an obvious spinodal in the variance curve of the inter-particle distances along the increasing cutoff distances, implying a legible transition pattern between the liquid-like and crystal-like aggregations. This work bridges the aggregation phenomena of physical particles and swarming of organisms in nature upon revealing some common mechanism behind them by slightly varying their inter-individual attractive/repulsive functions, and may find its potential engineering applications, for example, in the formation design of multi-robot systems and unmanned aerial vehicles (UAVs).

## Introduction

Collective behaviors of various kinds of self-driven particles have attracted more and more attention in recent years. One of the most remarkable characteristics of systems, such as a flock of birds, a school of fish, or a swarm of locusts, is the emergence of *ordered state* in which the particles form difference appealing patterns moving in the same direction [1]–[3] despite the fact that the interactions are merely of short range. Revealing the nature of aggregation patterns will find direct application in many relevant engineering systems, such as attitude alignment of satellite clusters, multi-agent formation control, sensor network data fusion, traffic systems and so on [4]–[9]. The forming rule of the patterns [8] can also help us to understand more deeply the social aggregation phenomena like escaping panic [10], [11], decease contagion processes, as well as the evolution of cooperation [12]–[14], and many other population behaviors in the society [15], [16].

A basic yet popular self-driven particles model was proposed by Reynolds [17], where three heuristic rules are prescribed, (i) *separation*: steer to avoid crowding and collision; (ii) *alignment*: steer towards the average heading; (iii) cohesion: steer to move towards the average position. These rules have been proven effective and are often used to describing the biological groups [18]–[20]. Later, Vicsek *et al*. [1] proposed a well-known collective behavior model where each particle tends to move in the average direction of its neighbors. With the increasing intensity of external noise, the system undergoes a remarkable transition from an *ordered state* to a *disordered state*. In recent years, the Vicsek model has drawn more and more attention from the physics, biology, engineering and social science communities [2], [3], [19], [21]–[27]. As two representative following works, Jadbabaie *et al*. [25] have proven that all the individuals should be jointly connected to guarantee the velocity synchronization, and Grégoire and Chaté [2] modified the Vicsek model by changing the way the noise is introduced, which simplified the phase transition from a second-order to a first-order one.

Apart from the motion synchronization investigation, other scholars turned to study more deeply into the nature of aggregation patterns [19], [28]–[33]. Enlightened by the mechanism of the inter-molecule force, Breder [28] proposed a simplified attraction/repulsion (A/R) model composed of a constant attraction term and a repulsion term inversely proportional to the square of the inter-agent distance, whereas Warburton and Lazarus [29] studied the effects on cohesion of a family of A/R functions. More recently, Gazi and Passino [19] derived another A/R model which is closer to the inter-molecule force function, and analytically proved that a stable ring-shaped pattern can be yielded in a finite time. Analogously, by using a linearized A/R model, Moreau [24] proved that the group will form a bounded circularly moving pattern if and only if there exists an agent connecting to all other ones, directly or indirectly, over an arbitrary time interval.

As another milestone of aggregation pattern exploration, Couzin [34] designed a Three-Sphere model by inserting an orientation area governed by the Vicsek model between the attraction and repulsion areas of the A/R model. With such a model, three typical types of collective behaviors, i.e., swarming, torus, and migration, are observed. Particularly, torus well explains the circular motion pattern among fish schools, ant groups, bacterial colonies and slime molds. By adopting Couzin's attraction/alignment/repulsive mechanism, Tanner *et al*. [35] proposed a centralized algorithm and a distributed one leading to irregular collapse and irregular fragmentation, respectively. Later, Olfati-Saber [36] developed a general framework for flocking, which adopts Newton's gradient descent law of motion and hence eventually yields to a regular lattice movement pattern. As the continuation work for Couzin's [34] and Olfati-Saber's work [36], Zhang *et al*. [37]-[39] incorporated predictive mechanisms into the above-mentioned two models to accelerate the aggregation procedure, and Liu *et al*. [40] designed a global synchronization method for a class of dynamical self-driven particle systems.

With the rapid development of the interdisciplinary collective behavior investigation, more complex patterns have been revealed by physicists and biologists. Vicsek [41] exhibited the universal patterns among many organisms as well as non-living objects. Later, Juanico [42] proposed a modified kinematic model which leads to several stellate patterns by changing the distribution of preferred pairwise length. Impressively, some new basic laws are found out these years that embody some essential aspects of coordinated behavior of various systems ranging from colonies of tissue cells, flocks of birds to collectively moving robots.

Based on the previous works introduced above, one can see that the A/R interrelation mechanism among the self-driven particles has played an essential role of forming and enriching the collective patterns in both living and nonliving multi-agent systems. However, there are still very few works on *revealing the quantitative relationship between the A/R function's variation and the evolution of collective dynamic patterns*, which is of great interest for physicists, biologists and system scientists and hence motivated our present study. Therefore, from the aspect of physics, we demonstrate in this paper that physical particle systems also show some resemblance to biological groups, which bridges the seemingly different procedures of them.

More precisely, we examine the dynamic pattern's emergence by modifying the A/R inter-particle interactions. A quite interesting phenomenon is observed that particles will aggregate into some liquid-like or crystal-like clusters depending on the cutoff distance (or the cutoff in short) of the A/R function which embodies the vision range of each particle. To understand this observation more deeply, we analyze the forming mechanism of these two distinct patterns by non-balanced statistical physical methods, and then find an apparent transition between the liquid-like and crystal-like patterns merely by slightly changing the cutoff distance of the A/R interaction function. This work reveals some common features behind the various aggregation phenomena of physical particles and biological groups, and may find its potential engineering applications, for example, in the design of multi-robots systems, multi-sensor networks and UAVs.

The rest of the paper is organized as follows. In Sec., we present a self-driven particle model governed by a general A/R function. The transition phenomenon between the liquid-like and crystal-like patterns is exhibited with statistical physical analysis in Sec. Finally, the conclusion is drawn in Sec.

## Methods

We consider a group of 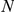 particles moving in a square shaped cell of linear size 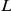 with periodic boundary conditions. The particles are represented by points moving continuously (off lattice) on the plane as below:
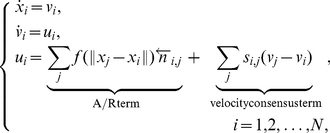
(1)where 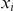, 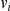 and 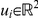 are the position, velocity and acceleration of the 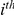 particle moving in a two-dimensional space, respectively, 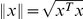 is the 2-norm, 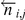 is a vector pointing from 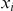 to 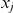 and 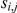 is the adjacent matrix (the definition will be given later) entries of the group's proximity matrix with
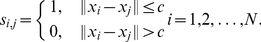
(2)where 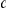 embodies the vision range of each particle, which equals the cutoff range of the A/R function. Beyond this value 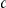 of inter-particle distance, the link between each pair becomes so weak that each particle will be invisible to the other. Therefore, we call any two particles 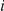 and 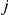 within Euclidean distance 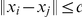 as an adjacent pair, and with such a definition, the whole group can be represented by a proximity network with nodes and edges representing the particles and the connections between the particle pairs. Note that the A/R term of the acceleration 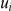 can be attraction or repulsion depending on the distance between each pair of particles inside the group.

In order to quantitatively study the role of interactions between particles, it is quite natural to seek assistance from the inter-molecule functions [43], [44], such as Lennard-Jones, Hard-Sphere, Square-Well and the six-ordered exponential potential models. Among these models, we adopt the Lennard-Jones potential [?] for its effectiveness in describing non-polar monatomic systems, and hence utilize a derivative exponential potential function and a second order polynomial to represent the attractive and repulsive interactions, respectively, as below,
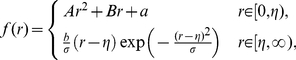
(3)where 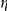 is the preferred distance between two particles. To guarantee the continuity and differentiability of the proposed A/R function (3), the function 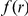 satisfies the following equations at the threshold 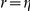:
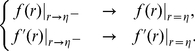
(4)


**Figure 1:**
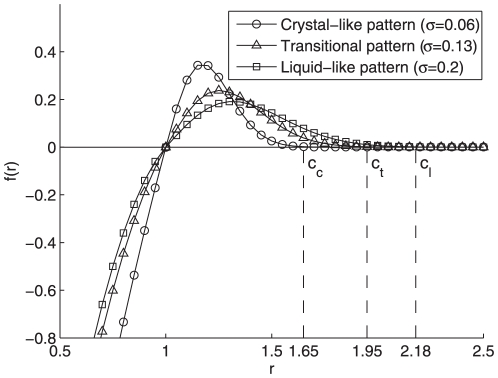
A/R function 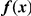 with different values of 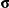. Here, the preferred distance 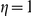, and 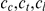 denotes the cutoffs of the crystal-like, transition and liquid-like patterns, respectively. It can be analytically proven that the vision range (or cutoff) 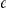 rises monotonously with increasing parameter value 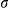 for fixed equilibrium 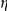.

One should not be intimidated by the six parameters 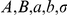 and 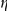 in the proposed A/R model since only two of them are free parameters under investigation. Let us explain this as follows. First, 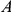 and 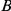 can be determined by the continuity and differentiability condition (??). Secondly, the effects of the parameters 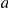 and 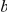 are much weaker than those of 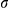 and 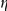, respectively. Thereby, without loss of generality, we set 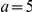 and 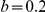 and focus on the effects of the essential factors 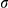 and 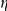 in the rest of the paper. To fulfill such a task, we demonstrate the A/R functional curves with different values of 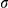 and fixed 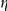 in Fig. 1. It can be analytically proven that larger parameter 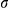 implies smaller peak value of 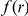, larger cutoff 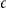 and longer settling time 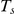. Therefore, the parameter 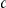 can be regarded as a vision range measurement of each homogenous particle as give in Eq. (2), beyond which the attraction vanishes. For example, if the vanishing threshold is 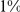 (i.e., beyond the cutoff 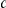, the attraction intensity is less than 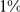 of its peak value), cutoff 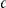 can be approximated as 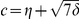. Regarding the other free parameter 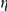, it represents the equilibrium distance between each pair of adjacent particles, or 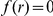 at 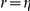, which is also essential to form different kinds of aggregation patterns.

## Results and Discussion

With the proposed model (3), we are now ready to investigate the role of A/R function on the forming and evolution of the collective motional patterns. In a two-dimensional 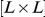 square with periodic boundary conditions, 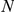 particles are initialized with identical velocities 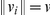. The initial locations and directions are randomly selected from 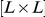 and 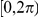, respectively. The dynamics of all the particles are updated every 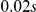.

**Figure 2:**
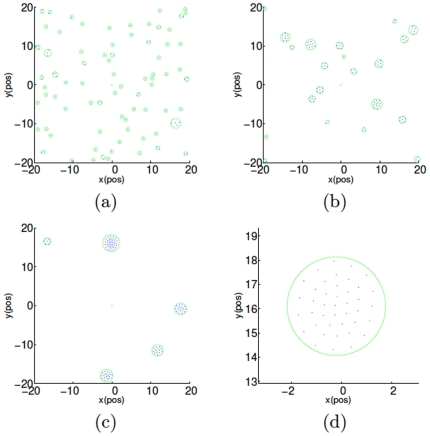
(Color online) Liquid-like pattern of 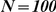 particles moving in a square-shaped cell with periodical boundary conditions. Here, 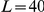, 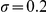, 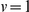 and 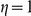. Subfigures (a), (b) and (c) are the snapshots at the 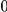
th, 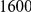
th and 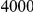
th running steps, and (d) shows the zoomed in liquid-like cluster or “drop”. In order to highlight the shape of the clusters or “drops”, we use green circles to mark their contours along the entire evolution. The initial locations and directions are randomly selected from 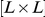 and 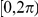, respectively.

**Figure 3:**
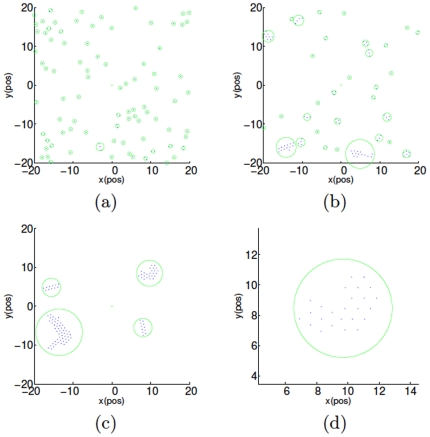
(Color online) Crystal-like pattern of 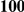 particles with 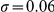. Subfigures (a), (b) and (c) are the snapshots at the 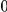
th, 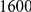
th and 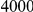
th running steps, and (d) shows the zoomed in crystal-like cluster. All the other settings are the same as Fig. 2.

**Figure 4:**
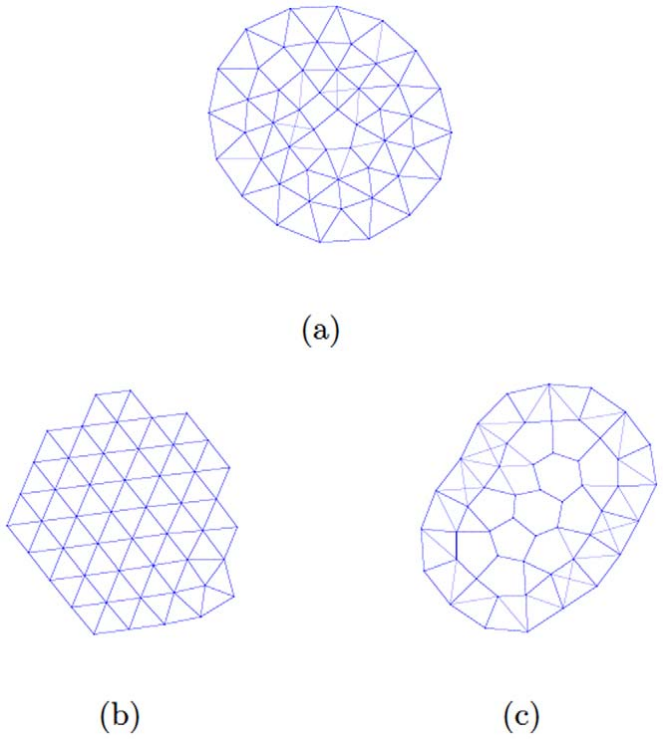
(Color online) Aggregation patterns with 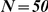. (a) The “liquid-like” pattern with 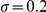. (b) The “crystal-like” pattern with 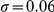. (c) The transitional pattern with 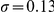. All the other settings are the same as Fig. 2.

A remarkable transition phenomenon from so-called *liquid-like* pattern to *crystal-like* one emerges in the numerical simulations along with increasing 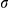 (see Eq. (3)). In detail, for the liquid-like pattern as shown in Fig. 2 and Fig. 4(a), some small clusters of particles are formed with structures quite similar to liquid drops among which the particle density is decreasing from the drop kernel to the surface due to the “surface tension”. Moreover, when multiple clusters or “drops” encounter, they will merge into a larger ring-shaped cluster or “drop” no matter what the original orientations and velocities the former “drops” were in. In comparison, for the crystal-like pattern, larger clusters are formed with much more evenly distributed particles as shown in Fig. 3 and Fig. 4(b), where a regular lattice-shaped formation emerges, which resembles molecules' distribution in crystal phase. When multiple crystal-like clusters encounter, the merged cluster will form an irregular shape determined by the original orientations and velocities of the previous clusters. Furthermore, the collective dynamics of the self-driven particles is more complex than these two aforementioned patterns, as there still exists a quasi-stable transient intermediate pattern [45] between them as show in Fig. 4(c). This pattern embodies a mixture of the crystal-like internal lattice together with the liquid-like ring-shaped external features. We call it a transient status since such a “partially melted” pattern is much weaker than the liquid- and crystal-like ones, whose corresponding range of 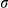 is much smaller than those of the two latter ones. Thereby, the dynamics of the self-driven particles is dominated by the liquid-like and crystal-like patterns, whose characteristics are the focus of our investigation.

Apart from the emergence of the three distinct patterns, it is also observed from Figs. 2 and 3 that the connectivity of the group's communication proximity net cannot always be guaranteed, which means that some particles will lose the connections with the others and hence the whole multiple particle group will always be separated into smaller clusters.

**Figure 5:**
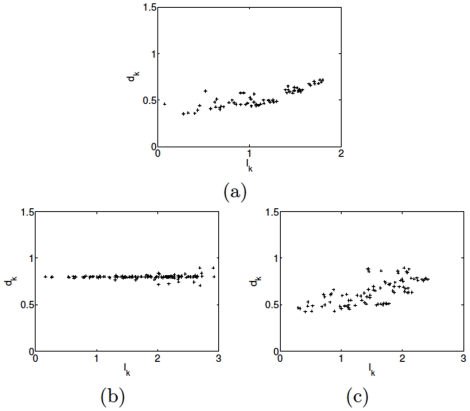
(Color online) The distance 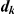 distribution with increasing 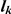. **Here**, the particle number 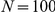 and 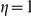. (a) “Liquid-like” pattern with 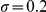, (b) “Crystal-like” pattern with 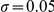, (c) Intermediate pattern with 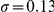. All the other settings are the same as Fig. 2.

To facilitate our investigation, we assume there are totally 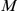 connections in the proximity net of the group, and then define 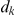 and 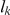 (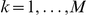) as the Euclidean distance of the 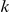-th link and the distance between the geometric center of each cluster and the middle point of 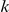-th link. With these definitions, we study the density's variation from inside to outside of each cluster by exhibiting the distribution of 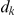 along with increasing 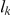 as shown in Fig. 5. Apparently, it is shown in Fig. 5(a) that 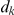 rises with increasing 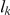, implying that the particles will become sparse from the kernel to the surface of each cluster, and hence this case corresponds to the liquid-like pattern. By contrast, Fig. 5(b) is self-consistent with the crystal-like pattern, where 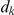 are independent of 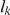 since the distance between each particle pair remains constant. Per the intermediate phase, Fig. 5(c) shows a mixture of crystal-like and liquid-like pattern, in which the neighboring distances 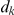
's also rise slightly with increasing 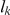. Nevertheless, the standard variance of 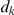 is much larger than those of the crystal-like and liquid-like phases, which well explains the irregular features of the “partially melted” phase.

**Figure 6:**
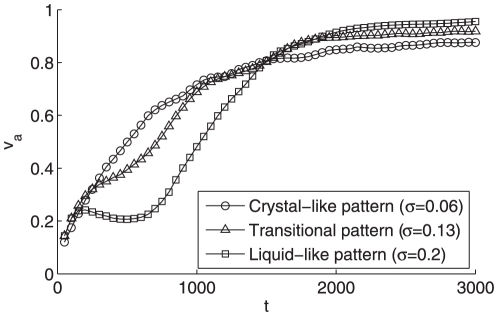
The average velocity 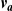 of particles in three patterns with 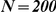 and 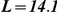. This curve is the average over 100 independent runs. All the other settings are the same as Fig. 2.

**Figure 7:**
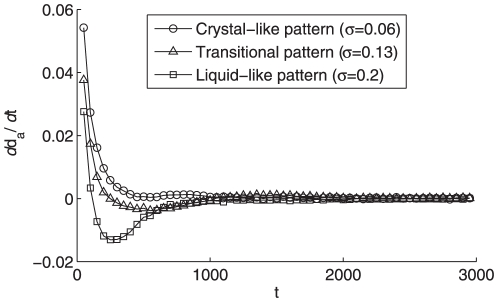
The evolution of the derivative of average neighboring distance 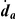 of particles in three states. All the settings are the same as Fig. 2.

In order to quantitatively analyze the dynamics of the different patterns, we adopt two indexes, namely 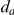 and 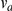, to measure the average neighboring distance and average velocity, respectively, as below,
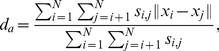
(5)

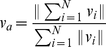
(6)with 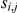 given in Eq. (2). Clearly, the value 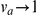 and 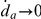 as the velocities of the particles achieve synchronization, so both 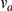 and 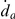 can be regarded as an *order parameter*. Note that 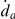 demonstrates the evolution of the average inter-particle distance, thus it contains more information than 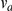 and we display both 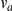 and 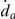 in Figs. 6 and 7, respectively. Indeed, due to the periodical boundary condition, the particles can communicated with the other ones for a sufficient number of times, and hence the particles in all these three patterns will eventually reach a synchronized velocity [18], which is also verified by Fig. 6. Moreover, it is also exhibited that the synchronization procedure of the liquid-like pattern is quicker than that of the crystal-like one. The underlying reason is that the former has larger individual vision scope and tighter clustering formation, which implies more connections in the proximity net who accelerate the consensus procedure [18].

**Figure 8:**
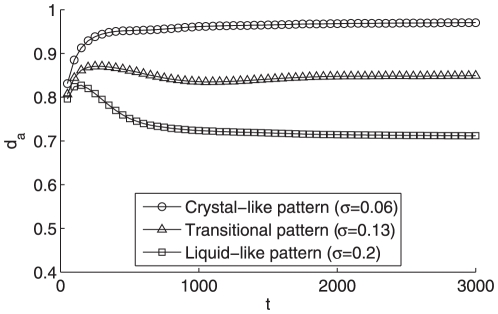
The evolution of average neighboring distance 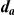 of particles in three states. All the settings are the same as Fig. 2.

One can understand more deeply about the dynamics of the system from the evolution of the average neighboring distance 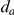 and its derivative 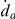 in Figs. 7 and 8, respectively. For the liquid-like pattern since the average distance 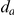 is much smaller (see Fig. 2(d)) than that of the crystal-like one (see Fig. 3(d)), its average distance derivative 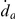 will experience a negative value during a long period until reaching a sufficiently small 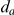 in Fig. 8, which well explains the negative overshooting of the liquid-like pattern in Fig. 7. Afterwards, its 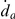 value settles down to zero quicker than that of crystal-like pattern due to its larger number of neighboring connections induced by larger individual vision scope and tighter clustering formation, which reveals the distinct forming procedures of the different aggregation patterns.

**Figure 9:**
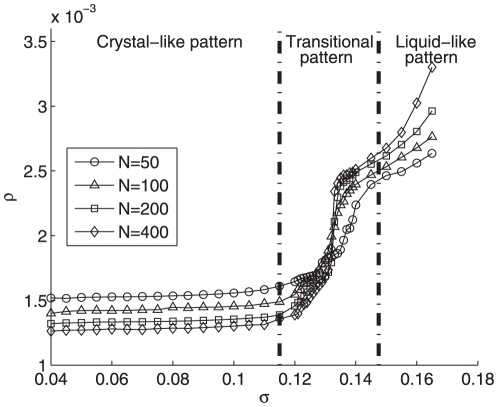
The particle density 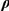 with 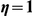 and particle number 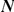 varying from 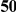 to 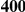.

**Figure 10:**
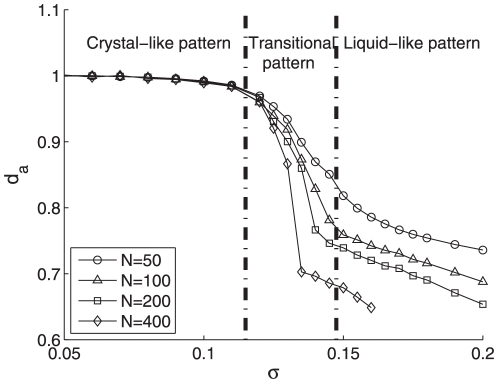
The average distance index 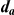 with 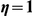 and particle number 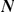 varying from 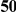 to 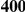.

To study the distinct features of the different aggregation patterns, we hereby demonstrate the density 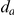 and average distance index 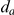 along with increasing parameter 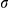 of Eq. (3) in Figs. 9 and 10, respectively. It is apparent from Fig. 9 that the particle density remains at a quite low level below 1.6 in the crystal-like pattern and then rises abruptly to the high lever over 2.3 representing the liquid-like pattern. Moreover, the intermediate range of 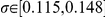 is so narrow that highlighting a clear pattern transition from the crystal-like pattern to the liquid-like one. Remarkably, in the crystal-like pattern as shown in Fig. 10, all pairwise distances remain constant roughly at 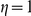. However, beyond a threshold of 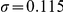, an evident declination of index 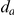 appears from about 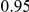 to around 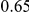 roughly at 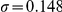, corresponding to the transient intermediate phase (see Fig. 4(c)) between liquid-like (see Fig. 4(a)) and crystal-like (see Fig. 4(b)) phases. Afterwards, the index 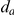 reaches a low level corresponding to the liquid-like pattern. Significantly, this intermediate region in Fig. 10 nicely matches the one of 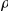 evolvement at Fig. 9, which strongly supports the existence of the transition from the crystal-like pattern to the liquid-like one.

Now, we are ready to derive that the dominating factor of the aforementioned three aggregation patterns is the cutoff distance 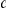 of the attraction interaction as shown in Fig. 1, which is measured by parameter 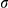 in Eq. (3). The physical rule behind such appealing phenomena can be summarized as follows. For a small cutoff 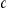, each particle will be attracted merely by the closest particles or neighbors. As a result, the distance between each neighboring pair eventually converges to an equilibrium value of 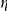 and hence particles will be evenly distributed in the aggregations clusters like regular lattices (see Fig. 4(b)). Conversely, for a sufficiently large cutoff 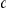, particles will be attracted not only by the adjacent particles but also by the ones far away. Consequently, the inner particles will be pressed closer to their neighbors whereas the outer ones enjoy larger separations as the pressure exerted on them is much weaker (see Fig. 4(a)), which eventually leads to a circular shape resembling liquid drops caused by surface tension.

Finally, due to its significance, we still emphasized the resemblance between forming procedures of the liquid-like pattern and natural liquid drops as below. In both cases, each particle round the kernel is pulled/pushed equally in every direction in 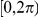 by its neighboring particles, resulting in a net force of zero. By contrast, the particles at the surface are mainly pulled inwards by other particles deeper inside the cluster, whose intensity is much less than that of the inner particles and is balanced merely by the group's resistance to compression. That is why the aggregation particle cluster forms a spherical-shaped liquid-like pattern with particle density decreasing from the inside to the outside.

### Conclusion

In this paper, we investigated the mechanism of the attraction/repulsion function of forming the different aggregation patterns of self-driven particles. In this function, the cutoff distance plays an essential role in the sense that, with a larger cutoff the particle aggregation shows a liquid-like pattern in which the outer particles are distributed sparsely while the inner ones densely. In comparison, however, when the value for the cutoff distance of attraction decreases to a sufficiently small value, the particle aggregation exhibits a crystal-like pattern as the distance between each pair of neighboring particles remains constant. An obvious spinodal or transient intermediate phase has been observed in the curves average inter-particle distances and the densities with respect to the increasing cutoff distance, indicating an evident pattern transition between the liquid-like and crystal-like aggregations.

From biological/physical interdisciplinary point of view, the contribution of this work lies in bridging the aggregation phenomena of physical particles and swarming of organisms in nature by revealing some common mechanism behind them. With such a revelation, our investigation helps to explain the natural aggregation pattern switching mechanism evolved by biological groups, e.g., during migration, an antelope herd generally forms like the crystal pattern or rigid lattice, but upon being attacked by predators, strong antelopes will quickly form a circle surrounding the weak one and hence the whole group will switch into the liquid-like pattern. From the aspect of engineering, designing different A/R functions for different aggregation patterns can be useful for various tasks like multi-robot and UAVs formation control. More importantly, this work bridges the forming procedures of phase patterns in both biological groups and physical substance's molecular clusters, which may brood more appealing findings in each area by seeking assistance from the relevant rules of the other one.
